# The Efficacy and Safety of Antipsychotic Medications in the Treatment of Psychosis in Patients with Parkinson's Disease

**DOI:** 10.1155/2016/4938154

**Published:** 2016-07-18

**Authors:** Nevena Divac, Radan Stojanović, Katarina Savić Vujović, Branislava Medić, Aleksandar Damjanović, Milica Prostran

**Affiliations:** ^1^Department of Pharmacology, Clinical Pharmacology and Toxicology, Faculty of Medicine, University of Belgrade, Dr. Subotića Starijeg 1, 11000 Belgrade, Serbia; ^2^Clinic of Psychiatry, Clinical Center of Serbia, Faculty of Medicine, University of Belgrade, Pasterova 2, 11000 Belgrade, Serbia

## Abstract

Psychotic symptoms are present in up to 50% of patients with Parkinson's disease. These symptoms have detrimental effects on patients' and caregivers' quality of life and may predict mortality. The pathogenesis of psychotic symptoms in Parkinson's disease is complex, but the use of dopaminergic medications is one of the risk factors. The treatment of psychotic symptoms in Parkinson's disease is complicated due to the ability of antipsychotic medications to worsen motor symptoms. The efficacy of clozapine in the treatment of psychosis in patients with Parkinson's disease has been confirmed in several clinical trials; however, the adverse effects and the necessity of blood count monitoring are the reasons why the use of this drug is challenging. The studies on safety and efficacy of other antipsychotics conflicting results. The use of antipsychotics in these patients is also associated with increased mortality. Psychotic symptoms in Parkinson's disease* per se *are also proven predictors of mortality. Thus it is necessary to treat psychotic symptoms but the choice of an antipsychotic should be based on careful risk/benefit assessment. Pimavanserin as a novel therapeutic option with more favorable adverse effects profile is now available for this indication, but careful postmarketing monitoring is necessary to establish the true picture of this drug's long-term safety and efficacy.

## 1. Introduction

Parkinson's disease (PD) is a complex neurobehavioral disorder. Among behavioral problems, psychotic symptoms (hallucinations and/or delusions) are highly relevant because they significantly affect the quality of life and complicate the treatment of motor symptoms by limiting the use of medications [1, 2].

Psychotic symptoms are present in up to 50% of patients with PD, but estimates vary widely [3–5]. They are usually associated with other correlates such as older age, more advanced age at the onset of the disease, duration of disease, disease staging, cognitive decline, presence of depression, and the use of antiparkinson medication [5–7]. Psychotic symptoms in PD patients are also associated with increased caregivers' stress, increased rates of nursing home placement, and poor clinical prognosis [1, 8, 9]. Therefore, psychotic symptoms in patients with PD require treatment. The reduction of antiparkinson medication is usually the first step but is usually ineffective and accompanied with worsened motor symptoms [1]. When reduction of antiparkinson drugs does not improve psychotic symptoms, antipsychotic agents should be considered [10].

The aim of this review is to summarize the recent reported results regarding the role of antiparkinson medication in the pathogenesis of psychotic symptoms and the efficacy and safety of antipsychotic drugs in patients with PD.

## 2. The Role of Antiparkinson Medication in the Pathogenesis of Psychotic Symptoms in PD Patients 

The pathogenesis of psychosis related to PD is currently viewed as a complex of medication- and disease-related factors. Among medication-related factors, the use of dopaminergic drugs is particularly considered as a significant risk factor for the development of these symptoms. The ability of drugs that enhance dopaminergic transmission, such as amphetamine, to induce psychotic symptoms and the blockade of dopaminergic D2 receptors as the mechanism of action of antipsychotic medications help support the dopaminergic role of pathogenesis in drug-induced psychosis in PD [10]. Also, historically, psychotic symptoms in PD patients were rarely described in treatment-naïve patients [4].

The causal relationship between levodopa and dopaminergic medications and psychosis has not been definitely established. The results of experimental and clinical studies are conflicting [11]. In an animal model of PD, Fox et al. confirmed the propensity of levodopa and dopamine agonists to induce psychosis-like behavior in primates [12]. However, in a clinical study by Aarsland et al., no correlation between the prevalence of psychotic symptoms and the type, duration, and dose of antiparkinson drug therapy was found [13]. More recently Rai et al. found no correlation between psychotic symptoms and the class, dose, or duration of antiparkinson medications in patients with PD [5]. Contrastingly, Papapetropoulos and Mash demonstrated that all antiparkinson drugs can induce psychosis in PD patients [14].

It is possible that direct dopamine agonists, such as ropinirole, have more ability to induce psychotic symptoms compared to levodopa, as demonstrated by Rascol et al. [15]. This was not confirmed in a study by Ecker et al., who showed significant correlation between psychotic symptoms in PD patients and the use of pergolide, but not ropinirole, pramipexole, or cabergoline (dopamine agonists). More interestingly, they showed that levodopa carries the lowest risk for the induction of psychosis [11]. Similarly, Goetz et al. showed that even large doses of levodopa did not induce hallucinations in PD patients who already had history of nightly hallucinations [16].

Although evidence is conflicting, antiparkinson medications seem to be a risk factor for the development of psychotic symptoms in PD. Apart from medication, it is now accepted that other, disease-related factors play significant roles in the pathogenesis of PD psychosis. Specific Lewy body disposition in ventral temporal regions of the brain, serotonergic/dopaminergic transmission imbalances, sleep dysfunction, genetic profiles, and visuospatial processing impairment may precipitate psychosis in PD [4, 10, 17, 18].

## 3. Antipsychotic Medications in Patients with PD

The use of antipsychotic drugs in patients with PD is complicated due to their ability to block dopaminergic D2 receptors which can induce dyskinesia and other extrapyramidal symptoms. Antipsychotic drugs differ significantly in their affinity towards D2 receptors. It is generally accepted that second-generation antipsychotics (SGAs) are safer in patients with PD due to their lower D2 antagonism but they also can cause extrapyramidal symptoms, although in lower rates in comparison with first-generation antipsychotics (FGAs) [19].

*Clozapine*, the SGA, was the first antipsychotic that was recognized as safe and effective for the treatment of psychotic symptoms in patients with PD [20]. However, the first randomized double-blind controlled clinical trial on safety and efficacy of clozapine in PD patients yielded negative results [21]. Yet, the doses used in this trial were similar to those in the treatment of schizophrenia and were up to 150 mg/d. Many open-label as well as double-blind trials conducted after that demonstrated clozapine's efficacy and safety [1, 22, 23]. A 2007 meta-analysis of randomized clinical trials that evaluated safety and efficacy of SGAs for this indication conducted by Frieling et al. confirmed significant improvement in psychotic symptoms with clozapine compared with placebo [24].

The efficacy and safety of extremely low doses of clozapine in the treatment of psychosis in PD patients were confirmed in two double-blind, placebo-controlled clinical trials [25, 26]. These doses are approximately tenfold lower than doses indicated in the treatment of schizophrenia. Current practice now is to introduce clozapine to PD patients in doses ranging from 6.25 to 12.5 mg/d and not to exceed 50 mg/d [27]. These doses dramatically reduce psychotic symptoms without worsening of the motor symptoms [1].

Other concerns regarding the use of clozapine in PD patients are anticholinergic effects, metabolic disturbances, and the risk of agranulocytosis. The first two phenomena are dose-related and are usually avoided in PD patients due to use of low doses [27–29]. Yet, clozapine-induced severe neutropenia, with a neutrophil count below 0.5 × 10^3^/*μ*L (agranulocytosis), may occur with the reported incidence up to 0.91% [30, 31]. The risk of neutropenia is highest during the first year of treatment and is considered to be not dose-related, but an idiosyncratic (type B) immune-mediated reaction [31, 32]. There is strict blood count monitoring required during treatment with clozapine. This means weekly blood draws during the first six months of clozapine administration, every other week for the second six months and after that every four weeks for the duration of the treatment, and also frequent visits to the pharmacy (“no blood, no drug”) [33]. This is quite challenging for the caregivers or health care providers, especially when the patients are in the nursing home settings. It is also reported as the main reason for the discontinuation of clozapine in nursing homes, even in patients with clear therapeutic benefits [34].

*Quetiapine*, which structurally resembles clozapine, showed conflicting results regarding efficacy and safety in the treatment of psychosis in patients with PD [35]. Initial open-label studies showed promising results, summarized by Shotbolt et al. [36]. However, two randomized, placebo-controlled clinical trials showed no advantage of quetiapine over placebo regarding efficacy, tolerability, and early discontinuation in patients with PD [37, 38]. In comparison with clozapine, in one rater blinded, prospective study by Merims et al. quetiapine was inferior regarding the reduction of the frequency of hallucinations but superior in reducing delusions [39]. In contrast to these findings, a double-blind, small sample, study by Fernandez et al. showed efficacy of quetiapine over placebo in reducing visual hallucinations in patients with PD [40], but in this study patients with delusions were excluded. Another randomized controlled trial of quetiapine in the treatment of psychotic symptoms in PD patients did not demonstrate efficacy of quetiapine [41], but the authors emphasized the limitations of the study (small sample size) and refrained from definite conclusions.

Despite lack of clear evidence for its efficacy, quetiapine is widely prescribed for the treatment of psychosis in patients with PD [36]. Its safety profile probably makes it an attractive alternative to clozapine, especially as quetiapine does not require vigilant blood count monitoring. In the abovementioned studies, quetiapine appeared generally well tolerated in PD patients, with the main reason for drop-outs being lack of efficacy, rather than adverse effects [10, 36]. Sedation and postural hypotension are the most frequently reported side effects of quetiapine in doses used in PD patients [42]. However, recent findings by Weintraub et al. showed increased mortality in PD patients treated with quetiapine, with hazard ratio of death compared to nonuse of an antipsychotic of 2.16. Even higher risks were shown for olanzapine and risperidone, but no results were shown for clozapine [43]. These results cast a shadow on the current attitude regarding safety of antipsychotics including quetiapine in the population of PD patients and highlight the need for cautiousness when managing psychosis in PD.

*Risperidone* is an SGA with adverse effects profile more similar to FGAs, especially regarding motor symptoms and hyperprolactinemia [19]. These effects are dose-dependent. Although PD patients with psychosis usually respond well to very low doses of antipsychotics, many clinicians refrain from prescribing risperidone to these patients believing it is poorly tolerated and might induce motor symptoms worsening [44]. The results of clinical trials on the efficacy and safety of risperidone in PD psychosis yielded conflicting results. Most of the trials are open-label and in general the efficacy of risperidone was demonstrated in all of them [10, 45–49]. In terms of side effects, the results are mixed. Ford et al. showed worsening of motor symptoms in 100% of the patients, while in a study by Meco et al. none of the patients experienced change in motor functioning, albeit in lower dose [48, 49]. In an only double-blind, randomized trial of risperidone and clozapine in the treatment of psychosis in PD, Ellis et al. showed similar efficacy of the two antipsychotics in reducing psychosis in PD but higher tendency of risperidone to worsen extrapyramidal symptoms [50]. With recent reports of risperidone increasing mortality in PD (hazard ratio of 2.46 over nonuse), it is clear that this antipsychotic should be prescribed to PD patients with extreme caution [43].

*Other antipsychotic agents*, such as olanzapine, ziprasidone, and aripiprazole, were also tried in patients with PD. Although initial results of the open-label trials were promising, olanzapine is now, after several double-blind studies, considered ineffective for the treatment of PD psychosis and not well tolerated due to motor symptoms worsening, even in low doses [1, 51–55]. It is also shown that olanzapine increases risk of death in PD patients with hazard ratio relative to nonuse of the drug of 2.79 [43]. Ziprasidone, due to its higher affinity for serotonergic receptors than for dopamine receptors, could be a reasonable option for the patients with PD. Early case studies and open-label trials without comparator suggested efficacy and acceptable adverse effects of ziprasidone in PD patients [56–59]. One case series study confirmed efficacy of ziprasidone in improving psychotic symptoms in PD, but side effects such as prolongation of  “off-periods” and pathological laughing that occurred in some patients were described as its possible side effects [60]. The results of a randomized, single-blind comparable trial of ziprasidone and clozapine demonstrated that ziprasidone is at least similar to clozapine in reducing psychotic symptoms in patients with PD [61]. Further double-blind randomized clinical trials are needed to establish efficacy and tolerability of ziprasidone in the treatment of patients with motor disorders. Aripiprazole is a very interesting antipsychotic with partial agonistic activity on serotonergic and dopaminergic receptors. In schizophrenia trials, it showed low propensity to induce extrapyramidal symptoms [44]. So far, the data on its efficacy in reducing psychotic symptoms in PD were variable. Several case reports and open-label studies reported favorable response in some patients regarding psychotic symptoms, but worsening of the motor functions was very common with this antipsychotic, leading to the conclusion that aripiprazole is not suitable for patients with PD [62–66]. 

*Novel Treatment Options*.* Pimavanserin* is a nondopaminergic, selective serotonin 5-HT2a inverse agonist, also devoid of affinity for adrenergic, histaminergic, or muscarinic receptors [67, 68]. Due to its unique mechanism of action in the heart's electrical cycle, pimavanserin is a viable treatment option for PD patients with psychosis since it does not worsen motor symptoms and does not cause sedation [67]. It has recently (April 2016) been approved by the Food and Drug Administration for the treatment of hallucinations and delusions associated with PD in the United States, thus becoming the first drug registered for treating psychotic symptoms in any of the movement disorders [69]. The efficacy of pimavanserin in the treatment of schizophrenia is currently being investigated [70]. Most common adverse effects of pimavanserin reported during placebo-controlled phase 3 clinical trial were urinary tract infections, falls, and peripheral oedema, but the incidence of these events was similar in the placebo group [67]. However, it has the potential to prolong QTc interval and therefore is contraindicated in patients with known QT prolongation or in combination with other drugs known to prolong QT interval [71]. Since pimavanserin is new on the market, rigorous postmarketing monitoring is necessary to establish the true picture of this drug's safety and efficacy.

## 4. The Adverse Effects and Mortality Risk Associated with Antipsychotic Treatment in Patients with PD

All antipsychotic medications have adverse effects. In PD patients, it sometimes may be difficult to distinguish between antipsychotic adverse effects and disease-related symptoms due to their overlap. In general, FGAs, by antagonizing D2 receptors in nigrostriatal pathway, induce significant movement disorders, such as bradykinesia, tremor, and rigidity [72]. FGAs also cause different degrees of sedation, anticholinergic side effects (such as constipation, urinary retention, or dry mouth), orthostatic hypotension, and hyperprolactinemia. These adverse effects are also associated with some SGAs, such as risperidone [19]. Therefore, FGAs and risperidone should be avoided in patients with PD due to their ability to aggravate Parkinson's symptoms. The use of SGAs in PD patients is more reasonable due to less risk of compromising motor functions. However, the safety profiles of SGAs differ significantly among representatives of this therapeutic subclass. Some general recommendations regarding use of antipsychotics in patients of older age apply to patients with PD as well. The majority of SGAs have cardiometabolic adverse effects, including weight gain, increased insulin resistance, dyslipidemia, and hypertension [73]. PD patients with diabetes, obesity, or dyslipidemia as comorbidities should not be prescribed clozapine and olanzapine, while clozapine, ziprasidone, and FGAs, as well as pimavanserin, should be avoided in patients with heart failure and QTc prolongation [74].

The use of antipsychotics is associated with increased mortality in PD patients [43, 75]. However, the psychotic symptoms in PD* per se *are also proven predictors of mortality [10, 76]; thus it is difficult to distinguish between the mortality risk associated with antipsychotics and mortality risk due to the severity of the PD. Some research data indicate that the introduction of SGAs reduced the mortality rate in PD patients with psychotic symptoms [77]. A recent study of the differences in mortality between PD patients treated with different antipsychotics and PD patients unexposed to antipsychotics showed that the use of antipsychotics is associated with more than double the hazard of death compared to nonuse [43]. The highest risk was seen with haloperidol, followed by olanzapine, risperidone, and quetiapine. The researchers in this cohort study matched the treated and untreated patients by all the relevant variables. However, the confounding by indication probably remains a limitation of this study, because it is possible that patients who required antipsychotic treatment had more severe and complicated forms of the PD, which could contribute to the increased mortality rates.

Although the treatment of psychosis in PD patients is associated with significant risks, these symptoms should not be left untreated because they have detrimental effects on patients' and caregivers' quality of life and may predict nursing home placement and mortality. It is essential to carefully measure* pro et contra* evidence for each antipsychotic for an individual patient with the aim of minimizing risk and maximizing therapeutic effect.

## 5. Conclusion

Treatment of psychosis in PD is a complex task. The only antipsychotic with clear evidence regarding efficacy in PD patients is clozapine; however, its possible side effects are the main reason why psychiatrists are reluctant to prescribe it to these patients. Also, it is often discontinued in nursing homes due to required blood count monitoring. Other antipsychotic drugs have not been proven effective and/or are accompanied with untolerable adverse effects. The increased risk of mortality is associated with the use of antipsychotics in PD and the untreated psychosis. Either nonpharmacological strategies or new pharmacological agents should be developed to meet the needs of patients with psychotic symptoms and PD. According to up-to-date evidence, pimavanserin is currently the most promising treatment option for these patients.
